# What topics caught your attention in 2017?

**DOI:** 10.1007/s12471-018-1093-5

**Published:** 2018-02-27

**Authors:** J. J. Piek

**Affiliations:** AMC Heart Center, Academic Medical Center, Amsterdam, The Netherlands

To evaluate the readers’ interest in specific cardiology topics the editorial board of the Netherlands Heart Journal needs to know which articles were downloaded the most in 2017. Moreover, it may also be relevant to you, the reader, to know which articles your colleagues considered useful for keeping up to date with the latest developments in our rapidly evolving cardiovascular field. The Netherlands Heart Journal is the scientific journal of the Dutch Society of Cardiology that offers the members the latest information on scientific activities in our centres. It is a free of charge, open access journal that our readers frequently use to download articles. The total number of downloads for the top ten articles in 2017 was more than 12,000 and covered the full spectrum of basic and clinical cardiology.

The top ten articles were seven reviews and three original articles indicating that our readers are particularly interested in brief summaries of literature on diverse topics to keep them updated for their daily clinical practice.

Because of the numerous recent studies on the use of novel anticoagulant agents (NOAC’s) and the duration of dual antiplatelet therapy, four of the ten articles concerned antithrombotic and antiplatelet therapy. In the review of Ten Berg et al. a personalised dual antiplatelet therapy approach is recommended in patients suffering from an acute coronary syndrome or undergoing percutaneous coronary intervention [1]. This is in line with the article of Damman et al. which summarises the comments of the Dutch acute coronary syndrome (ACS) working group on the 2015 European Society of Cardiology (ESC) guidelines for the management of patients with non-ST-segment-elevation acute coronary syndromes [2]. They conclude that the purpose of the ACS working group is to evaluate the implementation of the ESC guidelines into Dutch clinical practice. The review of Gimbel and Ten Berg also focusses on the management of non-ST-elevation acute coronary syndromes, but this time in a subcohort of elderly patients (≥70 years) [3]. The recommendations for management of this patient cohort during the in-hospital phase, as well as after discharge, are clearly summarised in this review. The article of Pisters et al. describes the real-life use of rivaroxaban in a Dutch subset of the XANTUS registry, showing the low rates of major bleeding and label-discordant dosing and high persistence rates during one year of follow-up of patients receiving rivaroxaban for non-valvular atrial fibrillation in a real-world setting [4].

The study of Abawi et al. describes the body mass index paradox and mortality after trans-catheter aortic valve implantation (TAVI) [5]. In this article, the authors report that TAVI is safe in different body mass index groups with respect to postoperative complication rates, but there appears to be an unclarified obesity paradox showing an improved survival after TAVI in obese and overweight patients.

In the article of Bergheanu et al. the authors summarise the current recommendations for the treatment of atherosclerotic disease emphasising that lowering low-density lipoprotein cholesterol with statin therapy remains the cornerstone of medical treatment [6]. In high-risk patients, the addition of cholesterol absorption inhibitors should be considered. The long-term efficacy and safety of the use of proprotein convertase subtilisin/kexin type 9 (PCSK9) inhibitors are awaited and so are the results of treatment with other novel lipid-lowering agents that are currently being tested in ongoing clinical trials.

The article of Van Opbergen et al. highlights the role of calcium sensitive pathways in arrhythmogenic cardiomyopathy, evaluated in experimental models [7]. It is expected that the promising results of these studies will be followed by clinical studies, in particular in patients with arrhythmogenic right ventricular cardiomyopathy. The article of Michels et al. focusses on the management of patients with hypertrophic cardiomyopathy. It describes the different stages of this type of cardiomyopathy and the future perspectives on research in this field of interest, emphasising the need for collaboration between involved disciplines in specialised centres to optimise patient care of this relatively common disease [8].

The article of Garay et al. describes the early experience with the novel Venus *p*-valve for percutaneous treatment of native outflow tract obstruction [9]. Preliminary results in 10 patients indicate the feasibility of pulmonary valve implantation in patients with tetralogy of Fallot, initially treated with the trans-annular patch technique. Finally, the article of Meier et al. highlights the usefulness of three-dimensional printing to replicate complex structures in congenital heart disease that can be used for educational purposes, pre-procedural planning and device testing to improve catheter-based therapy of adult patients with structural and congenital heart disease [10].
